# Differences in cause-specific mortality between healthcare workers and all other employees in Lithuania, 2011–2019

**DOI:** 10.1186/s12913-025-13006-y

**Published:** 2025-07-03

**Authors:** Povilas Kavaliauskas, Domantas Jasilionis, Audrius Dulskas, Evaldas Kazlauskas, Giedre Smailyte

**Affiliations:** 1 Department of Public Health, Institute of Health Sciences, Faculty of Medicine, Vilnius University, M. K. Čiurlionio Str. 21/27, Vilnius, LT-03101 Lithuania; 2 National Cancer Institute, Vilnius, Lithuania; 3 Max Planck Institute for Demographic Research, Rostock, Germany; 4 Demographic Research Centre, Vytautas Magnus University, Kaunas, Lithuania; 5 Institute of Clinical Medicine, Faculty of Medicine, Vilnius University, Vilnius, Lithuania; 6 Center for Psychotraumatology, Institute of Psychology, Vilnius University, Vilnius, Lithuania

**Keywords:** Mortality, Causes of death, Physicians, Healthcare workers

## Abstract

**Background:**

Healthcare workers face health risks, including stress, burnout, and communicable diseases, leading to higher mortality rates. However, excess mortality diminishes with better disease control and lifestyle factors.

**Methods:**

The study is based on the aggregated census-linked mortality dataset provided by Statistics Lithuania. The dataset was based on all 2011 census records, as well as death and emigration records from March 1, 2011, to December 31, 2019. The primary variable identified three groups: physicians, nurses and assistant nurses, and other healthcare workers. The reference groups consisted of individuals who were employed in all other sectors. We also conducted an analysis comparing highly educated healthcare workers to the highly educated workers in other sectors. The results are presented using age-adjusted sex-specific Poisson regression mortality rate ratios.

**Results:**

The four most common causes of death among healthcare employees were cancer, cardiovascular deaths, other causes of death, and external causes of death. Female nurses show significantly lower 0.86 (0.74–0.99) mortality due to malignant neoplasms than all other employees. Male physicians had lower mortality rates from smoking-related cancer (0.47 (0.24–0.95)); however, significantly higher mortality was found for digestive system diseases 6.29 (2.36–16.79) and liver diseases 5.1 (1.27–20.42). Highly educated male healthcare workers had 1.3–1.4 times higher all-cause, cardiovascular, and malignant neoplasm mortality than highly educated workers from all other sectors. Highly educated females working in health care had lower mortality for malignant neoplasms but significantly higher mortality for all other causes of death.

**Conclusions:**

Excess mortality due to digestive system diseases and alcohol-related causes of death among nurses and other health care employees is a particular matter of concern and should be addressed by appropriate prevention policies. Further in-depth studies on risk factors are needed to explain mortality differences between the groups of healthcare and other sector employees in Lithuania.

**Supplementary Information:**

The online version contains supplementary material available at 10.1186/s12913-025-13006-y.

## Introduction

Healthcare workers are a critical element in every society around the world. Working in hazardous conditions exposes this occupational group to numerous well-established (e.g., stress or burnout) and specific health risks, such as higher exposure to communicable diseases [1]. These disadvantages could lead to the perception that the mortality rate among medical professionals might also be higher. For example, one of the first pieces of historical evidence about physician mortality in England and Wales in 1860–1880 shows that if compared to the general male population, male physicians had higher death rates for all causes of death and for 23 out of the 27 listed causes of death [2]. For suicide deaths, the risk of dying for medical professionals was 1.5 times higher [2]. The excess mortality of physicians tends to diminish in time, to a large extent due to a better understanding and control of diseases and lifestyle factors [3].

Recent studies conducted in several European countries reveal that mortality risk among doctors became lower or the same as in the general population, except for suicide. A mortality study from Norway showed doctors had the same death rates as the general population, except for suicide rates, which were 1.7 times higher for men and even 2.9 times higher for women [4]. Overall, medical doctors had higher mortality rates than other graduates in Norway. The study from Denmark shows that the standardised mortality ratio (SMR) for medical doctors was lower than for other graduates for cancer, circulatory diseases, and other natural causes [5]. However, the SMR for suicide was still 1.6 times higher for males and 1.7 times higher for females. A study from Massachusetts (USA) indicates that healthcare workers had a slightly lower mortality rate from deaths of despair (violent and alcohol- or drug-related deaths) than all other workers [6]. The same study revealed a striking disadvantage of medical assistants and other healthcare support occupation groups, showing more than two times the disadvantage in the risk of dying from these causes of death [7].

Systematic evidence about cause-specific patterns and specific determinants explaining mortality differentials between various groups of health care workers remains scarce, especially for the Central and Eastern European region. Using unique regional mortality data based on the linkage between the 2011 census and death records for 2011–2019, this study covering the entire population of Lithuania systematically explores all-cause and cause-specific mortality differentials between the three big groups of health care workers (general physicians; nurses and assistant nurses; other health care employees) and the remaining employees employed in all other sectors. This study also contributes to the existing literature by exploring whether mortality differences also prevail between highly educated health care employees and the group of highly educated individuals employed in all other sectors.

## Materials and methods

### Study design

The study is based on the aggregated cross-sectional census-linked mortality dataset provided by Statistics Lithuania.

### Study population

**Fig. 1:**
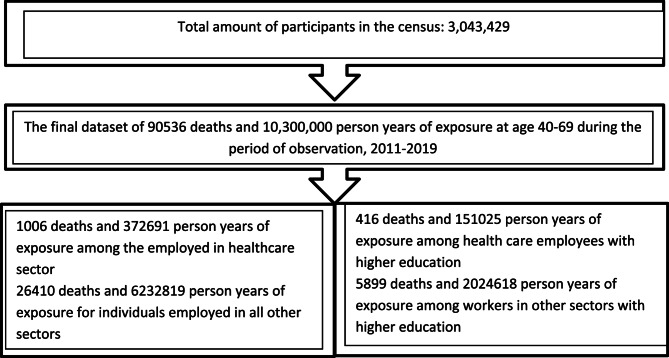
Flow diagram of participants included in the study

The aggregated dataset used for analyses includes cause-specific deaths and population exposures by all possible combinations of the following variables: age, sex, education, and occupation. The final dataset includes the total number of 1,082,805 individuals; out of them, 34,427 individuals were employed in the healthcare sector at the moment of the 2011 census. Figure 1 represents the flow of the individuals included in the final analysis.

### Inclusion and exclusion criteria

The study population is restricted to the ages 40–69 years for each calendar year of observation. The lower age band of 40 years was selected because information about occupation is fixed at the 2011 census baseline. Thus, for 40-year-old persons in 2019, information about their occupation comes from the 2011 census conducted 8 years ago – i.e., when these persons were approximately 8 years younger. Choosing a lower age limit is not feasible because of high occupational mobility at younger ages.

### Data sources

The dataset was built by implementing a longitudinal mortality follow-up using the baseline at the 2011 population census on March 1, 2011, and the end of the observational period on December 31, 2019.

### Data quality

All individuals at the census were followed from the census date until their death or emigration dates or until the end of the observational period. All data linkages were performed at Statistics Lithuania following data protection rules. Only anonymised data were provided for scientific purposes. The death registration is considered as 100% complete. Due to the fact that not all individuals immediately report their departure from Lithuania, emigration statistics may underestimate true emigration. However, we believe that undercounting emigration does not have any significant influence on our results because (a) most of emigration occurred below the age of 40 and (b) registration of emigration improved after introducing a special compulsory health insurance tax in 2010, which led to strong financial incentives to register departure from Lithuania.

### Study variables

The final total cause-specific deaths (dependent variables) and exposures were obtained by adding deaths, emigrations, and person-years lived within each calendar year and considering the changing age of all individuals during each calendar year. Independent categorical variables stem from the 2011 census and are fixed at the moment of this census (March 1, 2011).

### Study measures

Cause-specific deaths were classified using the 10th revision of the International Classification of Diseases (ICD-10) by the Institute of Hygiene. Due to the small number of deaths within groups of healthcare workers, only a few broad categories of causes of death were applied. The occupational variable of analysis identifies three large groups of health care workers being employed at the census, including (a) physicians, (b) nurses and assistant nurses, and (c) other health care employees. High educational status was identified using the self-reported information at the 2011 census and later classified using the International Standard Classification of Education (ISCED) 2011 [8] by Statistics Lithuania. Higher education refers to tertiary university or non-university education (ISCED categories) [5–8].

### Study outcomes

To assess the mortality differences between the three groups of healthcare workers and all remaining employees, a multivariate sex-specific Poisson regression was applied using the group of the employees (workers) employed in other than healthcare sectors as a reference category. The results based on models controlling for age are reported using Poisson regression mortality rate ratios (MRRs) and their 95% confidence intervals. In order to take into account possible differences in age structure across occupations, all Poisson regression models were controlled for age. The first analysis covered the entire population (the MRRs for unemployed and economically inactive are not reported), whereas the second analysis was restricted only to the individuals with high education. Additional sensitivity analyses on the distribution of age-standardised death rates for all causes of death are shown in the Online Annex 1. We performed statistical analyses using STATA 14.2 (Stata Corp., College Station, Texas, USA).

## Results

During the follow-up period between 1 March 2011 and 31 December 2019, the entire population aged 40–69 years experienced 90.5 thousand deaths, whereas 27.4 thousand deaths occurred among the employed individuals.

A detailed flow chart represents the total number of individuals, deaths and person-years of exposure in the final study (Fig. 1). 1006 deaths were registered among healthcare employees (291 deaths among physicians, 414 deaths among nurses and assistant nurses, and 301 deaths among other healthcare employees). The four most common causes of death among healthcare employees were cancer deaths (470 cases, 47%), cardiovascular deaths (236 cases, 23%), other causes of death (109 cases, 11%), and external causes of death (99 cases, 9.8%). 111 thousand deaths occurred among individuals with higher education; 6315 of them were employed. 416 deaths were registered among the healthcare employees with higher education.

The first analysis (Table 1) compares all-cause and cause-specific mortality in the three groups of healthcare employees to the group of the employed in all other sectors (a reference category). Male and female MRRs for all-cause mortality reported in Table 1 showed that there are no statistically significant differences between any of the three groups and the employed in other sectors. Yet cause-specific results revealed some peculiarities. Female nurses show significantly lower mortality due to malignant neoplasms than all other employees (reference category), whereas the corresponding male nurse and nurse assistant groups do not have such an advantage. Male physicians display more than 50% lower smoking-related mortality than in the reference category, whereas the remaining two male healthcare employee groups and all corresponding female groups do not show such a pattern (Table 1). At the same time, female physicians were the only healthcare employee group showing significantly lower mortality due to cardiovascular system diseases than in the reference category. The highest excess mortality (5–6 times) was found among nurse and nurse assistant males for digestive system and liver diseases. Albeit less pronounced, this disadvantage was also statistically significant for other healthcare employees. For females, the only healthcare employee group showing moderate excess mortality due to digestive system diseases was nurses and nurse assistants (Table 1). Other male healthcare employees also had a threefold mortality excess for alcohol-related causes of death. Finally, nurse and nurse assistant males maintained threefold excess mortality due to all other causes of death. The remaining differences were not statistically significant.

**Table 1:** Poisson regression mortality rate ratios for males and females at ages 40–69 years. 2011–2019. Reference group: employed in all other sectors

	Physicians and specialists				Nurses and assistant nurses				Other healthcare workers			
	Death counts	MRR	Confidence limits		Death counts	MRR	Confidence limits		Death counts	MRR	Confidence limits	
MALES												
All causes of death	148	0.97	0.83	1.14	13	1.30	0.75	2.23	65	0.96	0.75	1.23
All malignant neoplasms (C00–C97)	48	0.94	0.71	1.25	3	0.95	0.31	2.95	17	0.82	0.51	1.32
Smoking-related cancer (C00-C14, C32-C34)	7	**0.47**	**0.24**	**0.95**	2	1.90	0.47	7.60	5	0.73	0.30	1.75
All cardiovascular diseases (I00-I99)	61	1.05	0.82	1.35	3	0.81	0.26	2.52	26	1.06	0.72	1.56
Digestive system diseases (K00-K92)	7	0.78	0.37	1.64	4	**6.29**	**2.36**	**16.79**	**10**	**2.26**	**1.21**	**4.21**
Liver diseases (K70-K77)	5	0.93	0.38	2.23	2	**5.10**	**1.27**	**20.42**	**9**	**3.27**	**1.69**	**6.30**
All external causes of death (V01-Y98)	17	0.76	0.47	1.22	0	-	-	-	10	0.87	0.47	1.61
Alcohol-related causes (F10, K70, K74, X45, I42.6)	6	0.83	0.37	1.84	2	3.76	0.94	15.06	**11**	**2.94**	**1.62**	**5.32**
All other (remaining) causes of death	15	1.07	0.65	1.78	3	**3.13**	**1.01**	**9.73**	2	0.30	0.08	1.22
FEMALES												
All causes of death	143	0.87	0.74	1.03	401	0.94	0.85	1.04	236	0.96	0.85	1.10
All malignant neoplasms (C00–C97)	82	0.92	0.74	1.14	197	**0.86**	**0.74**	**0.99**	123	0.93	0.78	1.11
Smoking-related cancer (C00-C14, C32-C34)	6	0.81	0.36	1.81	14	0.76	0.45	1.30	12	1.13	0.63	2.01
All cardiovascular diseases (I00-I99)	24	**0.62**	**0.41**	**0.92**	79	0.84	0.67	1.05	43	0.78	0.58	1.06
Digestive system diseases (K00-K92)	9	0.89	0.46	1.73	41	**1.41**	**1.02**	**1.93**	21	1.28	0.83	1.99
Liver diseases (K70-K77)	6	0.91	0.40	2.03	28	1.39	0.95	2.05	11	0.98	0.54	1.79
All external causes of death (V01-Y98)	13	1.01	0.58	1.75	38	0.98	0.71	1.36	21	0.98	0.63	1.51
Alcohol-related causes (F10, K70, K74, X45, I42.6)	4	0.53	0.20	1.43	27	1.20	0.81	1.76	14	1.11	0.65	1.89
All other (remaining) causes of death	15	1.13	0.68	1.89	46	1.27	0.94	1.72	28	1.37	0.94	2.00

Bolded values are marked as statistically significant *p* < 0.05

The second analysis was restricted to the employed individuals with high education only and presents a comparison between highly educated individuals employed in health care and highly educated individuals employed in all other sectors (reference group). First, the results on all causes of death combined suggested a significant excess mortality among highly educated health care workers for males and not for females (Table 2). Second, cause-specific findings were somewhat unexpected and also differed by sex, with highly educated male healthcare workers showing 1.3–1.4 times higher mortality for malignant neoplasms and cardiovascular diseases. At the same time, highly educated female health workers had lower mortality for neoplasms and higher mortality for all other (remaining) group causes of death (Table 2).

**Table 2:** Poisson regression mortality rate ratios for highly educated males and females at age 40–69 years working in health care. 2011–2019. Reference group: highly educated and employed in all other sectors

	MALES				FEMALES			
	Death counts	MRR	Confidence limits		Death counts	MRR	Confidence limits	
All causes of death	174	**1.34**	**1.15**	**1.57**	242	0.95	0.83	1.08
All malignant neoplasms (C00–C97)	55	**1.34**	**1.02**	**1.75**	127	**0.83**	**0.69**	**0.99**
Smoking-related cancer (C00-C14, C33-C34)	9	1.20	0.64	2.26	10	1.01	0.53	1.94
All cardiovascular diseases (I00-I99)	70	**1.38**	**1.08**	**1.75**	45	0.89	0.65	1.20
Digestive system diseases (K00-K92)	13	1.73	0.99	3.03	15	1.20	0.70	2.06
Liver diseases (K70-K77)	9	1.91	0.97	3.76	10	1.29	0.67	2.49
All external causes of death (V01-Y98)	20	1.11	0.71	1.73	24	1.33	0.87	2.04
Alcohol-related causes (F10, K70, K74, X45, I42.6)	10	1.62	0.85	3.05	12	1.25	0.68	2.27
All other (remaining) causes of death	16	1.29	0.78	2.13	31	**1.48**	**1.01**	**2.16**

Bolded values are marked as statistically significant *p* < 0.05

## Discussion

This study contributes to filling the evidence gap on mortality differences between the healthcare workers and all other employees in Lithuania, a country in Central and Eastern Europe. Although no significant differences were found for all-cause mortality, focusing on specific causes of death reveals interesting and unexpected patterns. One of the matters of concern for health policies should be strikingly high excess mortality due to digestive diseases in the group of nurses and nurse assistants and excess mortality due to alcohol-related causes of death in the group of males working in the other health care sector. Importantly, physicians displayed lower mortality, especially in the cases of smoking-related cancers (males) and cardiovascular system diseases (females). Focusing only on the employees with high education also led to the identification of peculiar cause-specific patterns and notable sex differences. For example, highly educated males employed in the healthcare sector had a pronounced excess mortality for all causes of death, malignant neoplasms, and cardiovascular system diseases. At the same time, any similar excess mortality was observed for these causes of death for females. On the contrary, highly educated female healthcare workers had lower mortality for malignant neoplasms. However, highly educated female healthcare workers showed significant excess mortality for all other (remaining) causes of death. These cause-specific patterns should be investigated further in the future with more detailed studies.

Despite some specifics, outcomes of this study generally confirm common patterns found in other countries. For example, a study from the USA highlights the importance of education, which explains lower mortality in the group of healthcare workers [9]. Differently from our study, significantly lower all-cause, malignant neoplasm, and cardiovascular mortality in the group of doctors and other graduates was reported by Aasland et al. [4] One of the most striking facts reported in our study concerns persisting mortality excess of highly educated healthcare workers if compared to highly educated employees in all other sectors. This pattern contradicts the situation in the US, where white male physicians show higher survival rates than lawyers, all other professionals, or all men [7]. A similar pattern has been reported in Denmark [5]. One Norwegian study also found excess mortality among doctors which was explained by higher suicide rates [4].

Another important finding from our study concerns lower smoking-related mortality for male physicians. A 50-year observation study from Britain showed that non-smoking doctors survived on average 10 years more than those in the smokers’ group [10]. In addition to this, ceasing smoking at the age of 50 halved the hazards, and ceasing at 30 almost avoided all of them. A meta-analysis by Besson et al. showed that the prevalence of smoking among physicians was 21% [11]. Additionally, it was more common among male physicians and higher in Europe and Asia [11]. A study on smoking prevalence among university hospital staff in Lithuania showed that 37.6% of men and 9.7% of women were smokers. The highest prevalence of tobacco smoke was found among auxiliary personnel, men and women (44.9% and 11.3%, accordingly), and the lowest among doctors (36.4% and 7.6%, accordingly) [12]. In the general population, 41.4% of men and 11.3% of women were regular smokers [13]. Our study findings, to some extent, could be explained by lower smoking prevalence among health care workers in the past.

The results about excess mortality due to digestive system diseases are not consistent with those observed in other countries and can be attributed to a persisting unfavourable male mortality pattern in the former USSR countries. This finding might be considered as unexpected because physicians generally display lower mortality due to digestive tract mortality, due to a usually healthier lifestyle, reduced smoking rates and better healthcare access [14]. In a study by Aasland et al. [4], male doctors had lower digestive system disease death rates than the general population. Quite similar pattern was observed among the US doctors [7]. Meanwhile, our study not only did not find any such advantage for physicians but also reported on the strikingly high excess digestive system disease mortality among nurses and assistant nurses (both sexes) and other healthcare workers (males only). This pattern and indications of excess mortality (albeit not statistically significant in the group of nurses and nurse assistants in males) due to alcohol-related causes of death suggest a potential link to excessive alcohol consumption. Despite notable improvements since 2008, Lithuania remained the country with one of the highest global alcohol consumption levels and persisting heavy alcohol-related losses [15]. Although alcohol consumption has declined over the past decade—from 15.2 L per capita (age 15+) to 11 L [15]—mortality from alcohol-related causes remains among the highest in the European Union [16]. Prior studies reported that alcohol-related mortality is particularly high in lower education groups [17]. Therefore, a tendency of excess alcohol-related mortality among nurses and other health care employees found in our study might be connected to low-skilled occupations within these groups. In addition, increased and harmful alcohol use among healthcare employees may be associated with job-related chronic stress [1]. The role of mental health factors was highlighted in a Norwegian study, showing that excess mortality among doctors was attributable to a higher risk of suicide [4].

The importance of alcohol was also observed in other countries. For example, a study from Massachusetts [6] found that medical assistants, healthcare support staff, nursing, psychiatric, and home health aides had significantly higher mortality from alcoholic liver disease In contrast, physicians and surgeons had very low mortality. A large systematic review and meta-analysis by Wilson et al. [18] examined problematic alcohol use among physicians. However, the studies included in the review employed varying methodologies, complicating direct comparisons. Wilson et al. also noted that more recent studies suggest problematic alcohol use is more prevalent among female and younger physicians. However, patterns of alcohol use varied across different specialities and years of training.

The explanation of the observed mortality differences by specific causes of death requires representative studies on risk factors targeting health care employees. Unfortunately, to our knowledge, such evidence in Lithuania is very scarce or unavailable. Among the few studies, one study involving 238 physicians has been conducted on alcohol before and during the first years of the COVID-19 pandemic. The study reported about risky or hazardous alcohol use in 31.6% of male and 5.3% of female physicians [19]. In comparison, hazardous alcohol consumption was reported by 12.6% of French physicians [20] and 18.8% of Danish doctors [21], with risky alcohol consumption being strongly associated with burnout. There are only fragmentary and small-scale unrepresentative data about smoking prevalence and other CVD risk factors in Lithuania. For example, the 1999 study of the employees of the Kaunas Medical clinics (*N* = 3090) suggests that smoking prevalence was only about 13% [12]. The same study reported that the highest proportion of smokers was among the surgeons (42.7%). Unfortunately, there is no more comprehensive and more recent data comparing smoking among the employees in health and other sectors in Lithuania. When interpreting specific mortality patterns of healthcare employees in Lithuania, one should mention that health sectors in Central and Eastern Europe are over-represented by a larger number of employees working after retirement (beyond age 65 years) as compared to the situation in other sectors of the economy.

This study has several limitations. First, the employment and education status have been fixed in the 2011 census. However, we believe that introducing the lower limit of 40 years substantially diminishes the problem of possible changing occupation during the observational period. However, excluding young adults leads to a potential undercount of the role of specific problems among healthcare employees, such as alcohol consumption and external causes of death. Second, Lithuania’s relatively small population size did not allow for obtaining statistically robust mortality rate ratios even for the aggregated groups. It is possible that such a restriction artificially diminishes the real size of inequalities. Using a larger number and more detailed health care employment groups would provide more realistic and nuanced results about the distribution of group-specific mortality both within the health care sector and between the health care and all other employees. Third, our mortality data do not include any information about risk factors. Considering scarce information about the specifics of risk factors across employment sectors, the study provides very limited insights about potential determinants behind the observed mortality differentials.

## Conclusion

This study provides the first systematic evidence about differences in cause-specific mortality between the three groups of healthcare workers and employees in other sectors Striking excess mortality due to digestive system diseases and alcohol-related causes of death among nurses and other health care employees is a particular matter of concern and should be addressed by appropriate prevention policies. More targeted efforts are needed to produce comprehensive survey-based evidence on CVD and cancer risk factors explaining cause- and sex-specific patterns of mortality differences between the groups of health care and other sector employees in Lithuania.

## Supplementary Information

Supplementary Material 1.

## Data Availability

The original data used in this study were provided by Statistics Lithuania. Due to the agreement terms and data protection rules these data cannot be passed to the third party and should be requested directly from Statistics Lithuania. The STATA code used for analyses will be shared on reasonable request to the corresponding author.

## References

[1] Smith F, Goldacre MJ, Lambert TW. Adverse effects on health and wellbeing of working as a doctor: views of the UK medical graduates of 1974 and 1977 surveyed in 2014. J R Soc Med. 2017;110(5):198–207. 28504070 10.1177/0141076817697489 PMC5438063

[2] Ogle W. Statistics of mortality in the medical profession. Medico-Chir Trans. 1886;69:217–37. 10.1177/095952878606900112 PMC2121565 20896671

[3] Aasland OG. Physician suicide-why? Gen Hosp Psychiatry. 2013;35(1):1–2. 23123102 10.1016/j.genhosppsych.2012.09.005

[4] Aasland OG, Hem E, Haldorsen T, Ekeberg Ø. Mortality among Norwegian Doctors 1960–2000. BMC Public Health. 2011;11:173. 21426552 10.1186/1471-2458-11-173 PMC3070654

[5] Juel K, Mosbech J, Hansen ES. Mortality and causes of death among Danish medical Doctors 1973–1992. Int J Epidemiol. 1999;28(3):456–60. 10405848 10.1093/ije/28.3.456

[6] Kaki S, Hawkins D. Deaths of despair among healthcare workers, massachusetts, 2011 to 2015. J Occup Environ Med. 2021;63(6):449–55. 33683836 10.1097/JOM.0000000000002145 PMC8221712

[7] Frank E, Biola H, Burnett CA. Mortality rates and causes among U.S. Physicians. Am J Prev Med. 2000;19(3):155–9. 11020591 10.1016/s0749-3797(00)00201-4

[8] Schneider SL. The International Standard Classification of Education. 2011. In: Elisabeth Birkelund G, editor. Class and Stratification Analysis. Emerald Group Publishing Limited; 2013. pp. 365–79. (Comparative Social Research; vol. 30). [cited 2024 Jul 17]. Available from: 10.1108/S0195-6310(2013)0000030017.

[9] Olfson M, Cosgrove CM, Wall MM, Blanco C. Mortality Risks of U.S. Healthcare Workers. Am J Prev Med. 2025;68(6):1080–90. 10.1016/j.amepre.2024.11.005 PMC12092202 39612966

[10] Doll R, Peto R, Boreham J, Sutherland I. Mortality in relation to smoking: 50 years’ observations on male British Doctors. BMJ. 2004;328(7455):1519. 15213107 10.1136/bmj.38142.554479.AE PMC437139

[11] Besson A, Tarpin A, Flaudias V, Brousse G, Laporte C, Benson A, et al. Smoking prevalence among physicians: A systematic review and Meta-Analysis. Int J Environ Res Public Health. 2021;18(24):13328. 34948936 10.3390/ijerph182413328 PMC8705497

[12] Malakauskas K, Veryga A, Sakalauskas R. Rūkymo paplitimas tarp viešosios Gydymo Įstaigos Darbuotojų. Med (Mex). 2003;39(3):301–6. 12695645

[13] Buivydaitė K, Domarkienė S, Rėklaitienė OR, Tamošiūnas A. Vidutinio amžiaus Kauno Gyventojų Rūkymo Įpročių paplitimas, pokyčiai per 20 Metų Ir Sąsajos Su sociodemografiniais Rodikliais. Med (Mex). 2003;39(10):999–1006. 14578645

[14] Zimmermann C, Waldhoer T, Schernhammer E, Strohmaier S. Mortality of working-age physicians compared to other high-skilled occupations in Austria from 1998 to 2020. Scand J Work Environ Health. 2024;50(6):447–55. 10.5271/sjweh.4169 PMC11391937 38810246

[15] Alkoholio vartojimo paplitimas Lietuvoje - Narkotikų. tabako ir alkoholio kontrolės departamentas [Internet]. [cited 2024 Jul 13]. Available from: https://ntakd.lrv.lt/lt/statistika-ir-tyrimai/tendencijos-ir-pokyciai-lietuvoje/alkoholio-vartojimo-paplitimas-lietuvoje/.

[16] Mackenbach JP, Kulhánová I, Bopp M, Borrell C, Deboosere P, Kovács K, et al. Inequalities in Alcohol-Related mortality in 17 European countries: A retrospective analysis of mortality registers. PLoS Med. 2015;12(12):e1001909. 26625134 10.1371/journal.pmed.1001909 PMC4666661

[17] Pechholdová M, Jasilionis D. Contrasts in alcohol-related mortality in Czechia and lithuania: analysis of time trends and educational differences. Drug Alcohol Rev. 2020;39(7):846–56. 32909686 10.1111/dar.13157 PMC7756221

[18] Wilson J, Tanuseputro P, Myran DT, Dhaliwal S, Hussain J, Tang P, et al. Characterization of problematic alcohol use among physicians: A systematic review. JAMA Netw Open. 2022;5(12):e2244679. 36484992 10.1001/jamanetworkopen.2022.44679 PMC9856419

[19] Harmful use of alcohol among physicians before and during the first years of the COVID-19 pandemic| Journal of MEDICAL SCIENCES. J Med Sci| Medicinos mokslai. ISSN: 2345– 0592. Email: info@medicsciences.com. 2022 [cited 2024 Jul 19]. Available from: https://medicsciences.com/harmful-use-of-alcohol-among-physicians-before-and-during-the-first-years-of-the-covid-19-pandemic/.

[20] Thiebaud PC, Martin C, Naouri D, Le Joncour A, Truchot J, Yordanov Y. Alcohol consumption among French physicians: A cross-sectional study. Drug Alcohol Depend. 2021;218:108356. 33342514 10.1016/j.drugalcdep.2020.108356

[21] Pedersen AF, Sørensen JK, Bruun NH, Christensen B, Vedsted P. Risky alcohol use in Danish physicians: associated with alexithymia and burnout? Drug Alcohol Depend. 2016;160:119–26. 26832935 10.1016/j.drugalcdep.2015.12.038

