# Supplementary material for: Differences in cause-specific mortality between healthcare workers and all other employees in Lithuania, 2011–2019
Source: BMC Health Serv Res. 2025 Jul 3;25:914. doi: 10.1186/s12913-025-13006-y (PMC12224504; doi:10.1186/s12913-025-13006-y)
Supplement: Supplementary file 1 — Supplementary Material 1. [file 12913_2025_13006_MOESM1_ESM.pdf]

Differences in cause-specific mortality between health care workers and all other employees in Lithuania, 2011-2019

Povilas Kavaliauskas[1,2], Domantas Jasilionis[3,4], Audrius Dulskas[2,6], Evaldas Kazlauskas[6], Giedre Smailyte[1,2]
1. Department of Public Health, Institute of Health Sciences, Faculty of Medicine, Vilnius University, Vilnius, Lithuania; 2. National Cancer Institute, Vilnius, Lithuania; 3. Max Planck Institute for Demographic Research, Rostock, Germany; 4. Demographic Research Centre, Vytautas Magnus University, Kaunas, Lithuania; 5. Institute of Clinical Medicine, Faculty of Medicine, Vilnius University, Vilnius, Lithuania; 6. Center for Psychotraumatology, Institute of Psychology, Vilnius University, Vilnius, Lithuania

ONLINE ANNEX 1

Age-standardized* death rates (observed ASDRs per 100,000) and observed ASDR ratios (MRRs) for the employed in health care and employed in all other sectors, 2011-2019.

	**Males**		**Females**	
	**SDR**	**MRR**	**SDR**	**MRR**
**Analysis #1 (all employed)**				
Employed in all other sectors	745	1	272	1
Physicians and specialists	685	0.92	223	0.82
Nurses and assistant nurses	921	1.24	257	0.95
Other healthcare workers	709	0.95	260	0.95
**Analysis #2 (employed with higher education)**				
Employed in all other sectors	508	1	232	1
Employed in health care	671	1.32	212	0.91

* WHO European Population Standard 2013.
